# Factorial microarray analysis of zebra mussel (*Dreissena polymorpha*: Dreissenidae, Bivalvia) adhesion

**DOI:** 10.1186/1471-2164-11-341

**Published:** 2010-05-28

**Authors:** Wei Xu, Mohamed Faisal

**Affiliations:** 1 Department of Pathobiology & Diagnostic Investigation, College of Veterinary Medicine, Michigan State University, East Lansing, MI 48824, USA; 2 Department of Fisheries and Wildlife, College of Agriculture and Natural Resources, Michigan State University, East Lansing, MI 48824, USA

## Abstract

**Background:**

The zebra mussel (*Dreissena polymorpha*) has been well known for its expertise in attaching to substances under the water. Studies in past decades on this underwater adhesion focused on the adhesive protein isolated from the byssogenesis apparatus of the zebra mussel. However, the mechanism of the initiation, maintenance, and determination of the attachment process remains largely unknown.

**Results:**

In this study, we used a zebra mussel cDNA microarray previously developed in our lab and a factorial analysis to identify the genes that were involved in response to the changes of four factors: temperature (Factor A), current velocity (Factor B), dissolved oxygen (Factor C), and byssogenesis status (Factor D). Twenty probes in the microarray were found to be modified by one of the factors. The transcription products of four selected genes, DPFP-BG20_A01, EGP-BG97/192_B06, EGP-BG13_G05, and NH-BG17_C09 were unique to the zebra mussel foot based on the results of quantitative reverse transcription PCR (qRT-PCR). The expression profiles of these four genes under the attachment and non-attachment were also confirmed by qRT-PCR and the result is accordant to that from microarray assay. The *in situ *hybridization with the RNA probes of two identified genes DPFP-BG20_A01 and EGP-BG97/192_B06 indicated that both of them were expressed by a type of exocrine gland cell located in the middle part of the zebra mussel foot.

**Conclusions:**

The results of this study suggested that the changes of *D. polymorpha *byssogenesis status and the environmental factors can dramatically affect the expression profiles of the genes unique to the foot. It turns out that the factorial design and analysis of the microarray experiment is a reliable method to identify the influence of multiple factors on the expression profiles of the probesets in the microarray; therein it provides a powerful tool to reveal the mechanism of zebra mussel underwater attachment.

## Background

The Eurasian, non-native mollusk, the zebra mussel (*Dreissena polymorpha*), invaded the Laurentian Great Lakes in the 1980 s and has expanded since then to other regions in North America [1,2]. The spread of the zebra mussel has been followed by ecologic and economic devastation of unprecedented magnitude [3]. Through their unique ability to attach to underwater surfaces, the invading mussels interfered with navigation and flow of industrial effluents, a problem that has caused billions of dollars in losses [4,5]. Moreover, the selective filtering capacity of the zebra mussel has modified the microbial communities in the Great Lakes to the extent that some resident species have become endangered and others are facing extinction [3]. The problem is compounded by the fact that all control methods used to minimize the zebra mussel invasion impacts have been unsuccessful [6-8]. As a result, a pressing need has emerged to unravel the strategies used by the invading mollusk to survive and mechanisms governing its firm attachment to underwater surfaces.

The zebra mussel is unique among freshwater bivalves in that in the most of the life cycle, from post-veliger stage through adult stage, it keeps its byssus, a group of exocrine glands embedded in the mussel's foot that secretes threads with attachment pads through byssogenesis [9,10]. Except for the morphological characteristics, the biochemical nature of the byssus and processes involved in zebra mussel's adhesion to substrates remain largely unexplored. The byssus apparatus is considered to be a masterpiece of underwater bioadhesion because of the rapid and robust production, as well as the relatively simple components of adhesive proteins [11]. The silver hair-like structure that can be seen out of shells in most natural conditions is called byssal threads. All of the byssal threads are generated from the stem located at the root of the foot. The enlarged plaque end of the byssal thread is the structure holding all the adhesive proteins at the interface of the byssal thread and substrata surface. In the zebra mussel, the byssus also includes three main glands located in the foot, namely, the stem-forming gland, thread-forming land, and plaque-forming gland. The proteins needed for byssal thread formation and adhesion are secreted by the glands and expelled to the groove of the ventral side in the foot and transported out of the organ. The anatomic structure of the zebra mussel byssus and the production of the adhesive proteins have been well described by Rzepecki and Waite [9,10].

Three main protein components have been reported to be involved in the formation of zebra mussel byssal threads, namely, *D. polymorpha *foot protein (Dpfp) -1, -2, and -3 [9,10]. The preliminary studies on the Dpfp-1 and -2 suggested that both of these two foot proteins may serve as the component maintaining the byssal structures, rather than the adhesive proteins [12]. Dpfp-1 and Dpfp-2 contain a number of 3,4-dihydroxyphenylalanines (DOPAs) in their primary sequence which significantly strengthen the cuticle of byssal threads [13].

Our previous studies have identified 716 genes that are unique to zebra mussel byssus using the suppression subtractive hybridization (SSH) cDNA library technique [14]. Besides the Dpfp-1 and -2, a number of molecules with different putative functions have also been produced by byssus glands, such as the excretory gland peptides homologous to the salivary gland peptides of *Ixodes *spp. [15], host defense related molecules, and the proteins without predictable functions. The subsequently developed cDNA microarray with these 716 unique genes was used to compare the expression profiles of the gland unique genes under different attachment status. The results indicated that the expressions of 52 genes are either up or down regulated in the attachment status [16]. It is suggested that the microarray technique can be used as a high-throughput tool to study the mechanism of zebra mussel attachment at the molecular level.

Zebra mussel attachment is influenced by several environmental factors including water temperature [17], dissolved oxygen level [18], and current velocity [19]. The mechanism(s) by which these factors influence zebra mussel attachment is currently unknown and involves a large number of molecules. Identifying the influence of environmental conditions on gene encoding is rather complicated and may require a novel way of computation and statistical analysis. In this context, the studies of retinal development in zebra fish (*Danio rerio*) have adapted the multifactorial analysis to cDNA microarray data and proved useful in deciphering the complicated developmental process [20]. The factorial microarray analysis allows us to analyze the effects of more than one independent variable (the main factors involved in this study) on a dependent variable (the differentiation of gene expression) [21,22]. In this study, a non-reference loop experimental design was performed to test the effects of multiple environmental factors on the expression profiles of the byssus unique genes. This experimental design has been reported as a reliable design that leads to less disparity in precision and power comparisons between any two groups [23].

The purpose of the factorial design that we are going to describe here is to identify the influence of the temperature, water current velocity, dissolved oxygen, and byssogenesis status on the expression profiles of the molecules unique to zebra mussel byssus glands to thereby to better understand the mechanism of zebra mussel attachment under the complex underwater environment.

## Results

### Overview of the factorial analysis results

Microarray data analyses suggest that each of the factors tested (temperature, stirring, dissolved oxygen level and accessibility to attachment surfaces) had modified the expression of zebra mussel foot genes. The genes whose expressions had been affected by each of the four factors (*P *< 0.01) are listed in Table 1. The genes with 0.01 < P < 0.05 under the effects of experimental factors were all listed in Additional Files 1, 2, 3, 4.

**Table 1:** The genes whose expression profiles have been significantly modified by the change of experimental factors

Gene ID	Accession #	p.value	Log(FC)	Homologue
**Factor A (Temperature)**				
BG17_C09*	AM230384	7.00E-05	0.628	N/A
BG27_B08*	AM230019	0.00043	0.332	AAT92111.1| Excretory gland peptide NPL-2 [*Ixodes pacificus*]
BG28_H05*	AM229934	0.0052	-0.322	BAE93436.1| Shematrin-4 [*Pinctada fucata*]
BG03_B01*	AM229901	0.00653	-0.196	AAV80789.1| Excretory gland peptide [*Ixodes scapularis*]
BG23_D02*	AM229752	0.0068	-0.190	AAV80789.1| Excretory gland peptide [*Ixodes scapularis*]
Factor B (Water agitation)				
BG29_B09*	AM230251	0.00415	0.232	N/A
BG22_H06*	AM229789	0.00952	-0.156	N/A
Factor C (Dissolved oxygen)				
BG23_D06*	AM230109	0.0026	-0.270	N/A
BG14_F09*	AM230247	0.00639	0.16	N/A
Factor D (Attachment status)				
BG17_G02*	AM230231	0.00011	-0.6	N/A
BG10_C05*	AM229883	0.00114	0.146	AAV80789.1| Excretory salivary gland peptide [*Ixodes scapularis*]
BG33_H03*	AM230185	0.0013	-0.168	N/A
BG97/192_B06*	AM230076	0.00317	0.214	AAS92593.1| Excretory/secretory protein Juv-p120 precursor [*Litomosoides sigmodontis*]
BG20_F04*	AM230089	0.00339	-0.122	N/A
BG13_F10*	AM230013	0.00374	-0.130	AAV80789.1| Excretory gland peptide [*Ixodes scapularis*]
BG23_B03*	AM229897	0.00498	-0.184	AAV80789.1| Excretory gland peptide [*Ixodes scapularis*]
BG14_A11*	AM230170	0.00523	0.272	ABI52762.1| 60S ribosomal protein L27 [*Argas monolakensis*]
BG16_D03*	AM230168	0.00801	0.168	AAN05585.1| Ribosomal protein L22 [*Argopecten irradians*]
BG13_C11*	AM230205	0.00946	-0.124	N/A

When Log (FC) > 0,

In Factor A: the gene is up-regulated when water temperature is decreased;

In Factor B: the gene is upregulated when the mussel is under agitation;

In Factor C: the gene is upregulated when dissolved oxygen in water is at lower level;

In Factor D: the gene is upregulated when the mussel is under attachment.

While the gene expression profiles had been changed, however, the type and numbers of differentially expressed genes differed with each experimental factor used. At a 95% confidence level (*P *< 0.05), 73 genes (10% of the total genes on the slide) were differentially expressed by the accessibility of the mussel to attachment surfaces (S1). At the same confidence level, 27 genes (4% of the total number of genes) were found either up- or down-regulated with the temperature increased from 4°C to the room temperature of 22°C (S2). Twenty-six of the genes (4% of the total genes) were modulated by the level of dissolved oxygen (D.O.), which was close to that in the temperature group (S3). Surprisingly, only nine genes, 1% of the whole probesets on the array, were identified with their expression levels influenced by the current velocity (S4). With the *P *values set at 0.01, fewer probes were identified. Ten probes had their expression profiles modified by the status of attachment. Five genes demonstrated significantly different expression levels with the temperature change. With the level change, each of the other two environmental factors, D.O. and current velocity, modulated the transcription profiles of two genes only (Table 1).

**Figure 1:**
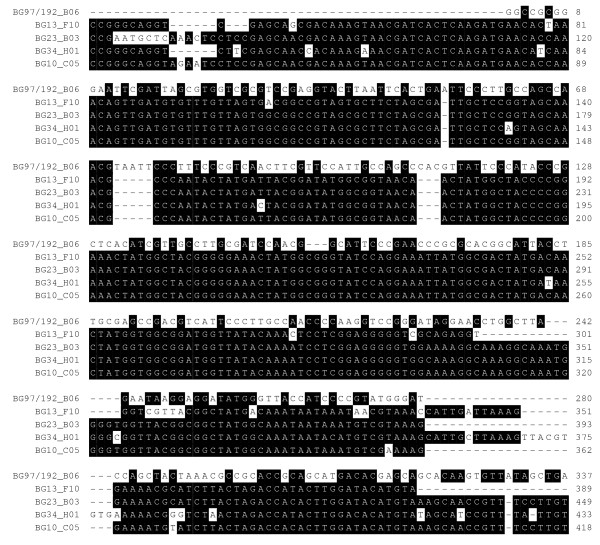
**Comparison of the primary structures of the excretory gland peptide encoding genes whose expression profiles can be modified by byssogenic activity**. The comparison was performed by using ClustalW multiple sequences alignment. The protein product encoded by the EST BG97/192_B06 is homologous to an excretory protein isolated from a filarial nematode *Litomosoides sigmodontis *while the rest of the EGPs encoded by selected ESTs have a salivary gland peptide from blacklegged tick (*Ixodes scapularis*) as their homologue. The nucleotides were colored with black when more than 50% nucleotides in this locus were identical. The gap between the nucleotides were labeled as "-".

**Figure 2:**
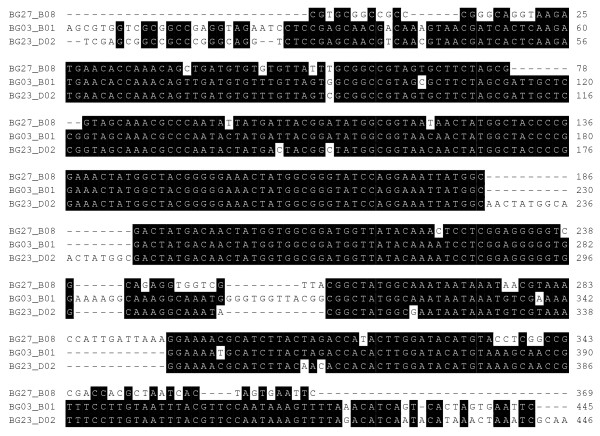
**The similarity of the EGP encoding genes demonstrated by multiple sequences alignment indicating the similarity**. The three EGP genes are all differentially expressed under the change of water temperature. The EST BG27_B08 is homologous to salivary gland peptide identified from the western black-legged tick (*Ixodes pacificus*). The other two probesets are all homologous to the blacklegged tick (*Ixodes scapularis*). The nucleotides were colored with black when more than 50% nucleotides in this locus were identical. The gap between the nucleotides were labeled as "-".

Among the genes that were modified by byssogenesis at the *P *< 0.01, five were homologous to invertebrate excretory exocrine gland peptides (GenBank accession number AAV80789 and AAS92593), while three genes from those modulated by temperature were homologous to two other invertebrate exocrine gland peptides (Accession numbers AAV80789 and AAT92111). The multiple sequences alignment suggested that the five genes in Table 1 whose expression was modified at *P *< 0.01 were not identical. BG31_H01 and BG10_C05 shared some similarities while BG13_F10 and BG23_B03 were structurally close to each other. The sequence BG97/192_B06 shared the least similarity to the other four genes in nucleotide sequences (Figure 1). Similarly, among the three genes from the temperature modulated gene group, BG23_D02 and BG03_B01 were closest in their primary structure and both of them were downregulated in response to the lower temperature. On the contrary, EST, BG27_B08 had its expression profile upregulated at low temperature (Figure 2).

### Validation

**Figure 3:**
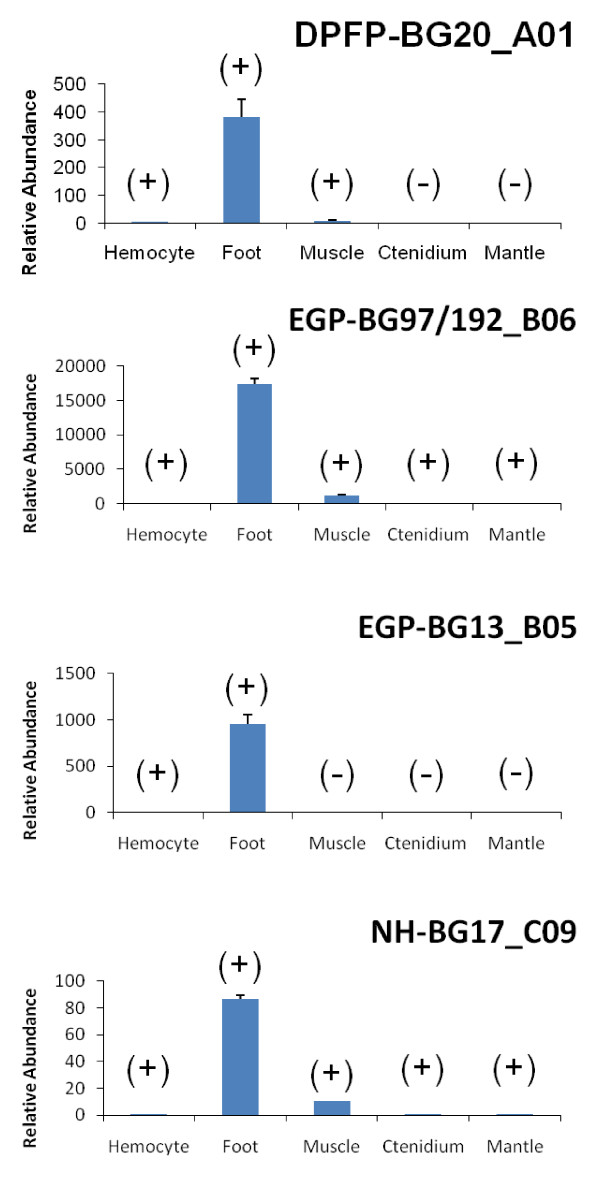
**The distribution of the mRNA products of the selected genes within zebra mussel tissues**. The (+) indicated the existence of the gene in the tissue; and the (-) suggested no detected transcripts in the tissue. For each gene, the lowest (+) sample was used as control and the relative expression levels of the gene in other tissues were calculated by using 2^-ΔΔCt ^model.

Four genes that were significantly modified by byssogenesis status were selected for validation. The qRT-PCR with RNA samples from five tissues of zebra mussels showed that the abundance of all four selected genes in the zebra mussel foot were extremely high as compared to other tissues. The RNA product of an exocrine gland peptide-like gene (EGP-BG97/192_B06) was detected in all selected samples; however, the expression level in the foot was significantly higher than in any of the others. The lowest expression level of EGP-BG97/192_B06 was in the ctenidium, which was 17399 ± 844 times less than that in the foot. Although the quantity of the gene EGP-BG97/192_B06 transcripts in the muscle was also higher than that in the ctenidium, the foot tissue produced 15 ± 1 times more gene product than the ctenidium cells. Another EGP gene, EGP-BG13_G05, was exclusively expressed in the foot and hemocyte while the ratio between expression levels in foot and hemocyte was 954 ± 56. The product of a Dpfp-like protein encoded gene, DPFP-BG20_A01 was detected in foot, muscle, and hemocyte samples. The abundance of the gene product in the foot was the highest, at 383 ± 63 times that in the hemocyte and 49 ± 8 times more than that in the muscle. Another gene that was expressed in all five tissues was a gene without known homologues in GenBank database, NH-BG17_C09. Compared to the least amount of RNA in the ctenidium, the foot provided 87 ± 3 times more NH-BG17_C09 product (Figure 3).

**Figure 4:**
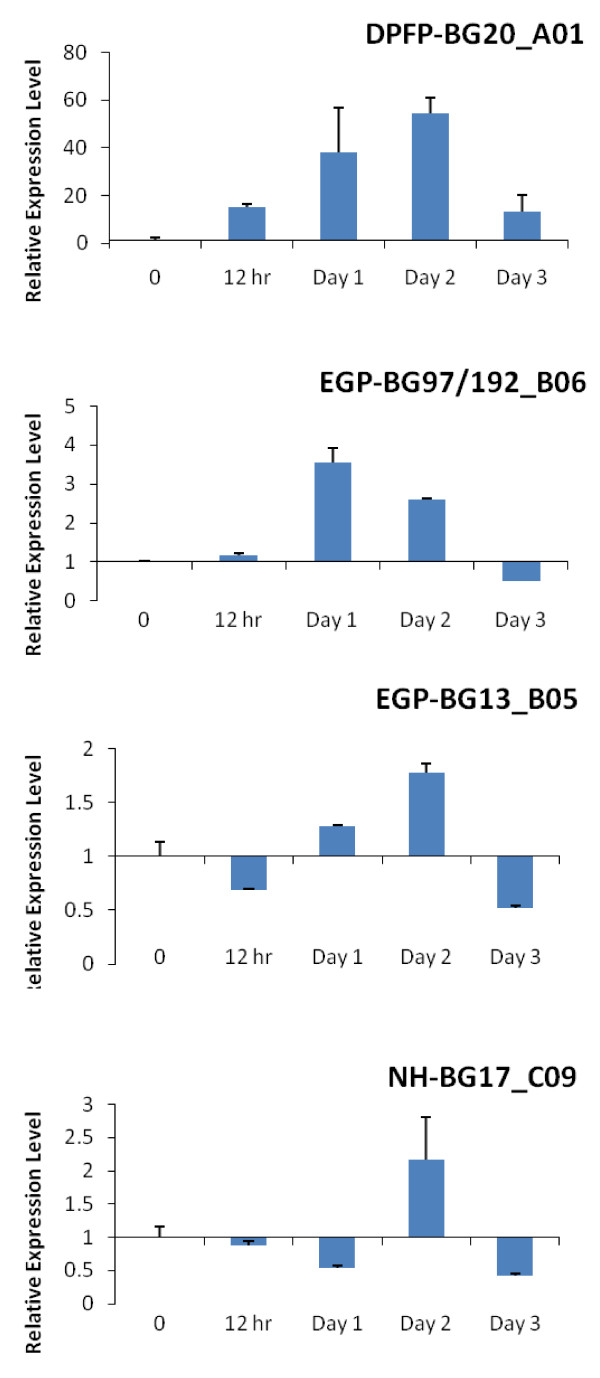
**The qRT-PCR results demonstrated the relative expression levels of the gene during the byssiogenesis**. The byssogenesis and non-byssogenesis samples were treated as described in materials and methods, and the non-byssogenesis sample was used as control with value 1. The amount of the transcripts of the gene was calculated by using 2^-ΔΔCt ^model. The comparisons of the gene expression levels between byssogenesis and non-byssogenesis and detached sample were made at 0, 12 hours, 1 day, 2 days, and 3 days post-treatment.

The comparisons of the expression levels of four selected genes between attached and detached status were also performed by qRT-PCR. As shown in Figure 4, compared to the detached mussels (D), the gene DPFP-BG20_A01 had significantly higher expression levels in attached groups (A) at 12 hours, 1 day, 2 days, and 3 days post-treatment. The most significant difference of expression levels between A and D was shown 2 days post-treatment. The two EGP-like protein encoding genes, EGP-BG97/192_B06 and EGP-BG13_G05, demonstrated similar expression profiles during the byssogenesis; both were upregulated in attachment status at 1 day and 2 days post-treatment, while down-regulated at 12 hours and 3 days post-treatment. However, the most obvious difference between A and D appeared at Day 1 in gene EGP-BG97/192_B06 and one day later in gene EGP-BG13_G05. At most time points, gene NH-BG17_C09 acted as downregulated in group A. Only at Day 2, the expression level of this gene demonstrated higher level in the attached status than the detached. All four candidate genes demonstrated the same expression trends to those identified by microarray assay.

### RNA *in situ *hybridization

**Figure 5:**
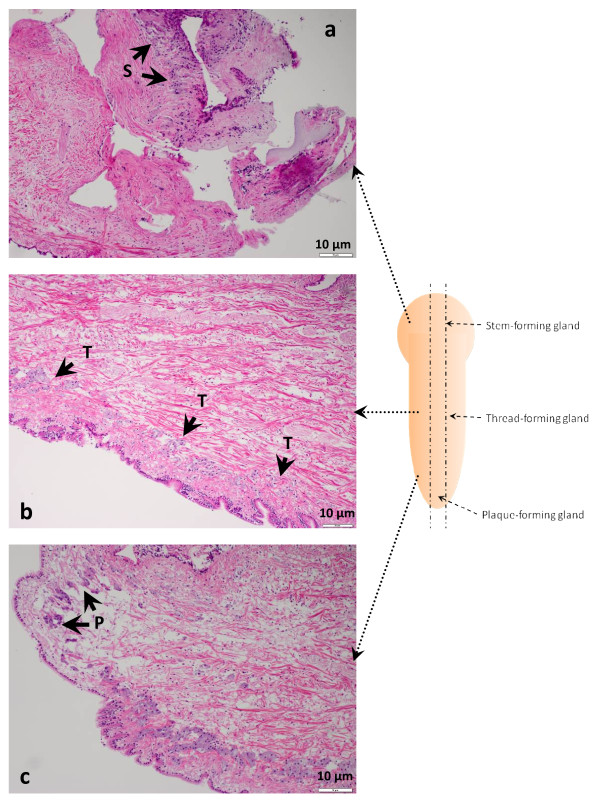
**The distribution of the zebra mussel byssus gland cells in mussel foot**. The zebra mussel foot sections are stained by H&E method. The sections are made along the longitude axis as demonstrated by the paralleled dash lines across the foot. Arrows with dashed line indicate the position of the three major byssal glands embeded in the mussel's foot. a: A longitudinal section in the root of the mussel's foot. Arrows point to the stem-forming glandular cells (S). b: The section of middle area of the foot. The light purple cells along the epethelial surface of the foot were thread-forming cells which were demostrated by arrows and the letter T. c: The tip of the foot containing the deep purple stained plaque-forming gland cells that were labled as P.

Three major byssus gland cells were observed from longitudinally sectioned zebra mussel foot with hematoxylin and eosin (H&E) stain; namely, stem-forming gland (SFG) cell, thread-forming gland (TFG) cell, and plaque-forming gland (PFG) cell (Figure 5). The SFG was located at the root of the foot containing the cells with the smallest size among the three types of gland cells (Figure 5a). The TFG cells were distributed in the middle section of the foot, along the epithelial cells on the surface. The color of the TFG cells was lighter than the other two types of gland cells, and the size of TFG cells was larger than SFG cells but smaller than PFG cells (Figure 5b). The cells of PFG had the largest size but the amount of this type of cells was the least and all the PFG cells were embedded in a small area of the foot tip (Figure 5c).

**Figure 6:**
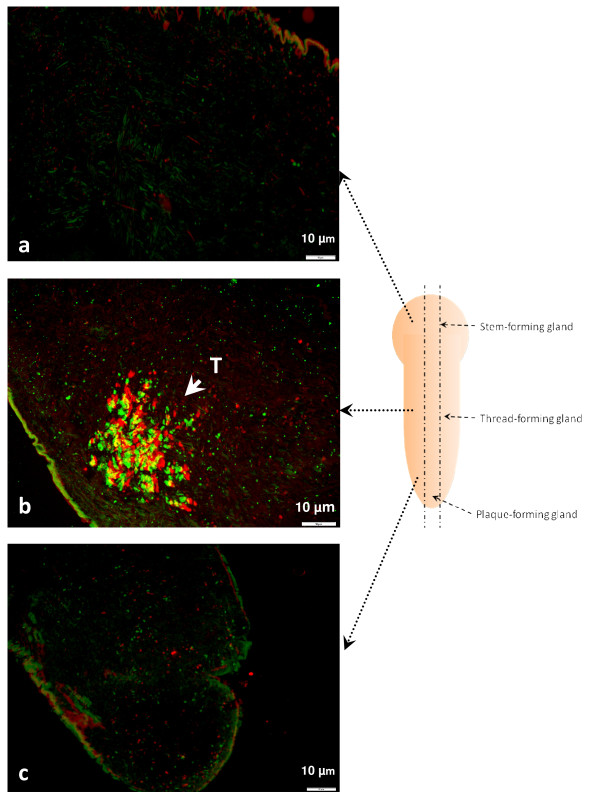
**The *in situ *expression of the gene DPFP-BG20_A01 and EGP-BG97/192_B06 in zebra mussel foot tissue**. The synthesized antisense RNA of DPFP-BG20_A01 was labeled with Alexa 488 dye (green) while the complementary RNA for EGP-BG97/192_B06 was labeled with Alexa 594 (red). The foot was cut along longitude as shown by the two paralleled dash lines across the foot. The positions of the byssus glands were labeled on the foot. The dash lines connected between slides and foot indicated the position of the three slides on the foot. a: The root area of zebra mussel foot including stem-forming gland; b: The middle region of the mussel foot containing thread-forming gland; c: The tip section of the foot including plaque-forming gland. T: Thread-forming gland cells.

The *in situ *hybridization results indicated that the transcription of both genes DPFP-BG20_A01 (Alexa 488 labeled) and EGP-BG97/192_B06 (Alexa 594 labeled) were not detectable at the foot root or tip sections (Figure 6a and 6c); however, strong signals of DPFP-BG20_A01 mRNA and EGP-BG97/192_B06 mRNA were detected in the middle area of the foot containing the TFG cells. The cells that expressed DPFP-BG20_A01 and those that contain EGP-BG97/192_B06 mRNAs were largely overlapped, demonstrating yellow signal. Some of the TFG cells that exclusively expressed either DPFP-BG20_A01 (green) or EGP-BG97/192_B06 (red) were also observed (Figure 6b).

## Discussion

The factorial analysis used in this study was designed as three enclosed loops encompassing four biological replicates for each treatment. This design allowed the estimation of the effects of the experimental factors with a minimal number of hybridized slides and a fairly high statistical power. In contrast to conventional microarray analysis, the factorial analysis is unique as it can properly estimate the potential effects of an experimental condition that was not directly included in the hybridization [20]. Most importantly, the factorial analysis of microarrays employed in this study is optimal for studies involving aquatic animals, as they are exposed simultaneously to a multitude of environmental factors that are in continuous fluctuation. Similar loop designs of microarray data were successfully applied by Wit *et al *[24] and Zou *et al *[25] and proved efficient in comparing gene expression modification due to multiple treatments. Despite the clear advantages of the factorial analysis of microarray data, the false discovery rate (FDR) continues to be a problem that cannot be ignored [26]. To minimize FDR, several statistical methods have been integrated into the microarray data analysis. In this study, we presented the data using both *P *< 0.05 and *P *< 0.01 as the cutoff for the microarray results. Subsequent validation demonstrated that many of the differential expression, of several genes with either *P *values in our study were indeed positive.

**Figure 7:**
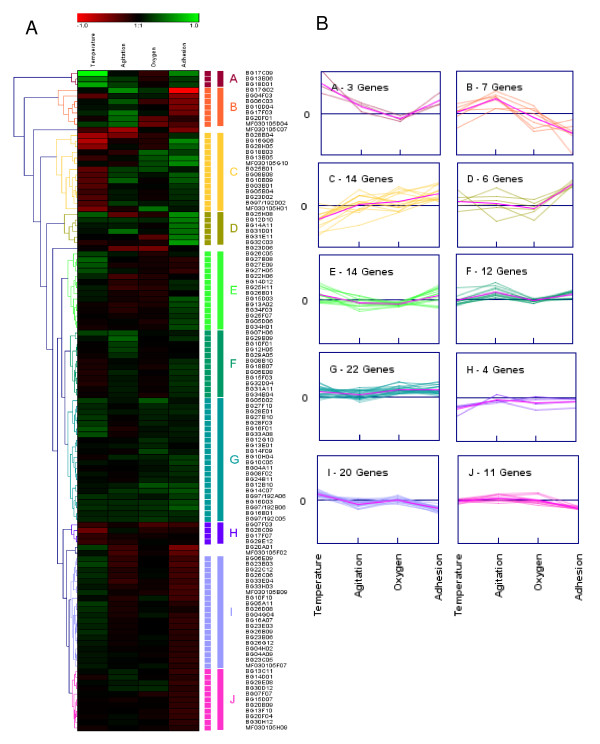
**The identified probesets with *P *< 0.05 were hierarchically clustered based on their expression profiles under the effects of the four factors**. One hundred and seventeen genes with differentially expressed profiles under the effect of at least one factor were used in this analysis. The logarithmic values of the ratios of two levels in each factor are indicated by different colors. Green and red color stands for up- and down-regulation, respectively. Black encodes no signifcant changes. (A) Hierarchical average linkage clustering. Ten clusters have been created with each of them labeled on the side of the heatmap. (B) The ten clusters represent the genes with the expressions induced by the change of the four factors.

**Figure 8:**
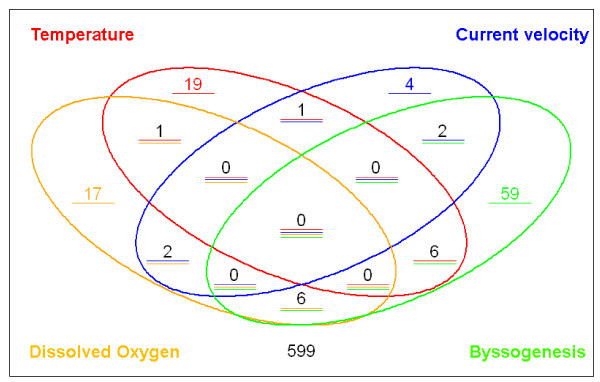
**The numbers of genes whose expression profiles are modified by single factor or multiple factors**. The values in the ellipse are the numbers of the probes which were modified by the factor with the same color. For example, the sum of the numbers in red ellipse is 19+1+1+6 = 27 meaning that 27 probes were found modulated by the factor A. The underline(s) under each number suggested the amount and types of factors could regulate its expression profiles. For instance, the number 19 with a read underline means the expression profiles of the 19 genes can only be affected by factor A while the value 1 with a red and blue lines suggests that one gene was regulated by both Factor A and Factor B.

However, the criterion for true positive selection indeed decreased the sensitivity of the microarray. Among the genes we selected for validation, some of them had their *P *values between 0.01 and 0.05. The qPCR results suggested that their expression profiles were also truly affected by the status of attachment although these genes were considered as false positive based on our criterion. Therefore, it is worthwhile to look in to the genes with 0.01 < P < 0.05. Ten major clusters were generated using the hierarchical clustering method done by the software Genesis [27] on the probesets whose expression profiles were modulated by at least one of the factors (Figure 7A). The genes in each cluster had similar expression patterns under the effects of the four factors. For instance, three genes had been grouped into cluster A, suggesting that the expression profiles of the three genes were significantly upregulated when the temperature was decreased or when the mussel was undergoing byssogenesis. Similarly, cluster B contained seven genes whose expression patterns were upregulated by water flow, through were downregulated by byssogenesis (Figure 7B). Among the genes identified by microarray analysis, some genes demonstrated dramatic expression level changes in response to the combination of more than one factor (Figure 8). For example, from the 59 genes affected by the attachment status (Factor D), six genes responded to changes in temperature (Factor A); another set of six genes were affected by dissolved oxygen levels (Factor C); and two other genes were affected by water current velocity (Factor B). Similarly, two genes were regulated by both Factor C and Factor D, while only one gene was affected by the combination of Factor B and C or Factor B and D (Figure 8).

Among the complete probesets identified by this array study, 62% were affected in response to adhesion status (Factor D), therefore, subsequent study of individual probes focused on this group of genes that were most probably associated with the zebra mussel's ability to attach. The four candidate genes selected for qRT-PCR were from this category. It was confirmed by qRT-PCR that after 48 hour treatment, the expression levels of these four genes were all upregulated during byssogenesis; however, curves of the relative expression levels of the four genes during the three-day byssognesis were not consistent. It is likely that these genes are involved at different stages during byssogenesis and are not always up- or downregulated. This periodical expression or depression may reflect the sequence of events of byssogenesis. Based on what has been observed in previous studies, within 12 hour post attachment, the temporary byssal threads are rapidly produced. Starting from 24 hours post attachment, permanent byssal threads become dominant [28]. The genes differentially expressed at these time points are very likely to be involved in the production of temporary or permanent byssal threads.

One of the identified genes, DPFP-BG20_A01, that had been validated by qRT-PCR is homologous to the Dpfp1, which was previously identified as an important protein for byssal thread structure [9,10] and was located by immunohistochemistry along the ventral groove of the zebra mussel foot [12]. The *in situ *hybridization performed in this study located DPFP-BG20_A01 in an area along the groove of the mussel's foot that is dominated by thread forming glands [29]. It is noticeable in this study that the expression profiles of many Dpfp-1 homologues were found in response to the change of adhesion status. This suggests that multiple foot proteins are involved in the byssogenesis of the zebra mussel in addition to the Dpfp-1. A number of foot proteins produced by byssus glands and playing different roles in the byssogenesis of Mytilids, another genus of mussels with a lifelong byssus apparatus, have been found over past decades [30-36]. It is very likely that, in the zebra mussel, there is also a group of foot proteins serving in different stages of byssogenesis.

Not only were foot proteins identified, but also the homologues of exocrine gland peptides (EGP) [37] were commonly found to be differentially expressed during byssogenesis. The transcription of one of the EGP-like molecules, EGP-BG97/192_B06, was also observed in the TFG cells area. This observation underscores the importance of these molecules in zebra mussel adhesion and that byssogenesis in the zebra mussel involves a myriad of proteins. The primary sequence of the EGP-BG97/192_B06 suggests its distinction from Dpfp-1, despite the domination of its amino acids with proline, tyrosine, aspartic acid, lysine, threonine, and glycine residues, which is similar to Dpfp-1 [38]. However, according to Francischetti *et al*, the homologous genes encode for salivary gland peptides with a variety of functions in ticks, such as anti-coagulant, anti-microbial, and oxidant metabolism [15]. In our analysis, the multiple sequences alignment with zebra mussel EGP-like genes in response to the byssogenic activities suggested that these molecules were structurally similar but not identical to each other. Therefore, their potential functions during byssogenesis are most likely different. It suggested that during the adhesion a number of molecules with various functions were involved in this process. Some of the molecules may be directly involved in the generation of the byssal threads and adhesive proteins, while others may play alternate roles in the complicated process such as protecting the byssal proteins from degeneration by microbes, releasing the adhesive proteins, and modification of byssal thread components.

Interestingly, more than one probeset in our data were homologous to Dpfp-1 or EGP encoding gene. However, the result of sequence alignment analysis on these sequences suggested that these sequences did not belong to a single Dpfp-1 or EGP. It has been reported that Dpfp-1 has 10-15 variants in natural conditions and this is a very typical structure for DOPA proteins, which are the main proteins in zebra mussel plaques to maintain the dove-tail structure of adhesive matrix [9]. It is very likely that the multiple Dpfp-1 homologues existing in our database are the different variants encoded by Dpfp-1 encoding gene; however, there's no evidence suggesting that EGP also has variants. If this hypothesis is also turned out to be true, the EGP can be also used by zebra mussel as another important matrix protein to sustain the adhesion structure in the plaque.

Multiple Dpfp-1 and EGP like protein coding genes were also identified in our previous study on the gene expression profile of zebra mussel during the byssogenesis [39]. Although sharing same homologues, these molecules may be involved in different process during byssus activities. For example, the transcripts of Dpfp-1 like protein encoding gene (BG15_F03-DPFP) was found in plaque-forming gland cells in previous study, while in this study we localize another Dpfp-1 like probe (DPFP-BG20_A01) in stem-forming gland. It indicated that these two Dpfp-1 homologues were involved in the activity of plaque and stem of byssus gland respectively [39]. Similarly, the transcripts of two EGP like protein encoding genes were also found differently distributed in zebra mussel foot [39]. Therefore, further study the homologues of Dpfp-1 and EGP is an essential step toward understanding the regulation of zebra mussel byssus glands.

The results of the study demonstrated that that very few genes had their expression profiles affected by more than one experimental factor. This suggests that each of the experimental factors affected only a few zebra mussel genes involved in byssogenesis independently; i.e., no uniformal response to all factors exists. A series of experiments was done by Clark and McMahon in 1996 to analyze the effects of temperature [17], hypoxia [40], and current velocity [19] on byssogenesis rate under laboratory conditions. The change of the water temperature seems to have the most significant effect on byssogenesis rate. From 5°C to 30°C, every 10°C shift caused dramatic difference in the rate of byssal thread regeneration. Even 5°C increase led to a significant increase of the byssogenesis rate when the temperature was above 25°C [17]. Our results demonstrated that the number of differentially expressed genes in response to temperature changes was the highest of the four factors tested (27 genes). On the contrary, the byssogenesis rate of the zebra mussel showed the least sensitivity to the change of current velocity. Only in a certain flow speed (0.2 m/s) was the byssogenesis rate of zebra mussel significantly higher than that in the other speeds (0.1 m/s, 0.15 m/s, 0.27 m/s) [19]. Consistently, the least number of genes (9 genes) identified by microarray showed the significantly differential expressions during the change of the current velocity. Comparing the genes significantly affected by the four experimental factors, we can easily see that adhesion status, temperature, and dissolved oxygen level tends to affect the byssogenesis by modulating similar genes. For example, the homologues of Dpfp-1, EGP, *C. elegans *neuropeptide-like proteins (NP_504109 and NP_505834), and oyster shematrins (BAE93436) [41] were widely identified. It is possible that the temperature and oxygen level along with the adhesion status, affect the byssogenesis through the similar pathway, while the current velocity seems to control this process through a different pathway.

For the genes that were modified by multiple experimental factors, it is also very interesting to observe how environmental factors can affect gene expression in different directions. For example, BG23_B03 is downregulated in attached status, while is upregulated with the decrease of the temperature; while BG97/192_B06 can be upregulated in the attached status and downregulated by the dissolved oxygen level. What these gene-environmental factor interactions mean to zebra mussel attachment and associated mechanisms, cannot be easily answered, primarily due to the fact that the putative functions of these genes remain largely unknown. In conclusion, the factorial analysis of the microarray results provides important information on how their expression is influenced by important environmental factors. This newly generated knowledge will help to better understand the molecular mechanism of zebra mussel underwater adhesion.

The factorial analysis of zebra mussel adhesion mechanism has provided for us a great tool to learn about the role of the four experimental factors during the zebra mussel attachment under controlled laboratory conditions. One can easily speculate that in the mussel's complex aquatic habitat more genes are likely to be involved in the attachment process. Additional studies, also using cDNA microarray factorial analysis, deem necessary to decipher the mechanisms governing zebra mussel byssogenesis, a process that has devastated the fragile ecosystem in infested waters of the Laurentian Great Lakes basin.

## Conclusions

Among the four factors tested in this study, the status of zebra mussel byssogenesis had the most obvious effect on the gene expression profiles of zebra mussel byssus genes. The other three factors, related to environment, also modulated the expression patterns of certain genes. Among the three environmental factors, the change of the temperature caused the differential expression of the most number of genes followed by the dissolved oxygen level in the water. The least number of genes were differentially expressed in response to the change of current velocity. This is highly consistent to the previous study on the byssogenesis rate influenced by environmental factors. Moreover, none of the genes were found with expression patterns affected by more than two experimental factors, which suggested the specificity of the byssus unique genes in response to the effects of the different factors. Produced by the zebra mussel thread-forming gland cells, the genes encoding DPFP-like protein and EGP-like protein are probably heavily involved in the zebra mussel byssogenesis in natural conditions.

## Methods

### Zebra mussel collection and maintenance

Zebra mussels used in this study were collected from Vineyard Lake in Brooklyn, MI, USA (Latitude: 42°4'59"N; Longitude: 84°12'34"W). The mussels were thoroughly cleaned and rinsed with deionized water several times before they were allowed to acclimate in aerated, filtered Vineyard Lake water for eight weeks. The mussels were kept in a glass tank and fed weekly with a pure culture of the algae *Ankistrodesmus falcatus*.

### Treatments of zebra mussels and experimental design

Three environmental factors were involved in this study including temperature (Factor A), current velocity (Factor B), and dissolved oxygen level (Factor C). The status of byssogenesis was considered as Factor D. The Analysis of Variance (ANOVA) model was performed on the expression values of each gene to represent how different factors contribute to observed values. Main factors and interactions of the experimental factors were included in the model. The model used in this study was:

This model includes the overall mean of gene expression (*μ_g_*), the main effects coefficients (*A*, *B*, *C*, *D*), the two-way interactions (*AB*, *BC*, *CD*, *AC*, *BD*, *AD*), the three-way interactions (*ABC*, *BCD*, *ABD*, *ACD*), the four-way interaction (*ABCD*), and the error term (*ε*). In each effect, two levels were created. To create two different attachment statuses of the mussels, we severed the byssal threads from randomly selected mussels and put them back into the tanks in different orientations. The mussels lying on the ventral side of their shells for 48 hours were considered to be attached individuals with byssogenic activity (A) while the ones with ventral sides facing up for 48 hours were taken as detached mussels without byssogenesis (D). Two temperature levels were used in this study: room temperature (R) and a low temperature level at 4°C. The two dissolved oxygen (D. O.) levels used were the normal D. O. level (N), which was maintained by airstones at about 10 mg/L, and the lower D. O. level, which was about 5 mg/L without airstones. The current of the water was performed by using VWR 371 Hotplate/Stirrer (VWR International LLC, West Chester, PA) and a magnetic stirring bar with a stirring speed of 60 rpm (F). The static water was used as non-current condition of Factor B in the experiment (S).

**Figure 9:**
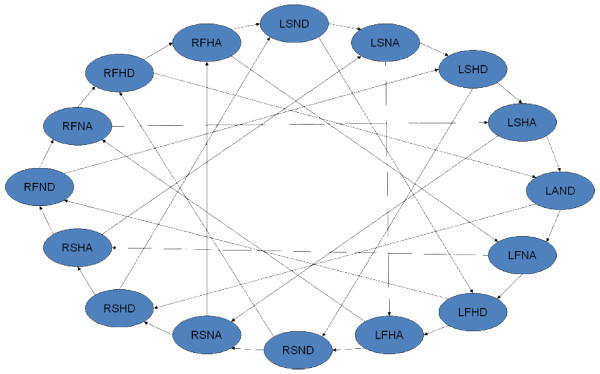
**Effects of temperature, dissolved oxygen, current velocity, and byssogenesis status on gene expression in the zebra mussel foot using cDNA microarray analysis**. The figure displays the different treatment combination selected as per the loop design approach. Each arrow represents one microarray hybridization. The start of the arrow stands for the sample labelled with dye Alexa 647 while the end point of an arrow represents the sample labelled with dye Alexa 555. L stands for low temperature (4°C) while R stands for a higher temperature (22°C); S stands for static water while F means flow water stirred by magnetic stirring bars; H represents the low dissolved oxygen level (5 mg/L) while the N represents the normal dissolved oxygen level (10 mg/L); A and D stand for attachment and detachment status, respectively.

Sixteen treatment combinations were obtained from this 2 × 2 × 2 × 2 factorial experimental design. To maintain high statistical power for the analyses of each factor effect, a loop experimental design was performed with four biological replicates for each treatment (Figure 9) [23]. Every two samples connected with an arrow were used to hybridize with one microarray slide. The dye labeling and hybridizations are indicated by Figure 9. Four null hypotheses were tested and the interpretation of the rejection of each null hypothesis is listed in Table 2.

**Table 2:** The null hypothesis and the interpretation of tested probesets.

Null hypothesis	Interpretation when null hypothesis is rejected
*H_0_*: *A *= 0	The expression profile of certain genes is modified in response to the surrounding temperature.
*H_0_*: *B *= 0	The expression profile of the gene is modified by the change of water movement.
*H_0_*: *C *= 0	The expression profile of the gene is modified by dissolved oxygen level.
*H_0_*: *D *= 0	The expression profile of the gene is modified by byssogenic activity.

### RNA extraction and cDNA synthesis

The total RNAs used for microarray hybridization were extracted from pools of zebra mussel feet with six individuals in each pool. Each treatment has four pools as four biological replicates. Therefore, the total number of zebra mussel used for each treatment is 6 × 4 = 24. The total RNA purification kit, 5-PRIME PerfectPure RNA Tissue Kit (5 PRIME Inc, Gaithersburg, MD) was used for RNA purification and the protocol followed the manufacturer's instructions. Total RNAs of all samples were quantified by Qubit^® ^Quantitation Platform with the Quant-iT™ Broad Range RNA Assay Kit (Invitrogen, Carlsbad, CA). The integrity of RNA was detected by 2100 Bioanalyzer (Agilent Technologies, Santa Clara, CA).

The RNAs were then reverse transcribed to single strand cDNAs by using SuperScript *Plus *Indirect cDNA Labeling Kit (Invitrogen) containing Alexa Fluor 555 & 647 fluorescent dyes. The cDNA syntheses and dye labeling procedures were all followed as described by the kit's instructions.

### Microarray hybridization and data analyses

The zebra mussel byssus cDNA microarray was developed and printed in the Center for Animal Functional Genomics, Michigan State University [16]. The cDNA microarray was designed based on the 750 genes obtained from a normalized cDNA library, which included 3% foot protein genes, 37% exocrine gland peptide encoding genes, 1% host defense related gene, 6% housekeeping genes, and 53% unknown genes [14].

The GeneTAC HybStation (Genomic Solutions) was used as the hybridizer for all hybridizations and an 18-hour step-down protocol was applied as described in our previous study [16]. The hybridized dual-channel array slides were scanned by GenePix 4000B two-laser Scanner (Molecular Devices, Downingtown, PA), and GenePix Pro 6.0 (Molecular Devices) software was then used for image processing and spot intensity file generation. The spot density files were analyzed with the Limma software package [42]. The preliminary data processing includes background correction, within array normalization, and between array normalization. The differentially expressed genes were determined by least square regression. The coefficients obtained for the experimental factors were based on the "sum-to-zero" parameterization. The logarithmic value of fold change of the gene expression under the effect of each factor was estimated by double the coefficient of each factor.

The false discovery rate (FDR) of the microarray used in this study was determined with a method described in our previous study [16]. Briefly, two cDNA samples synthesized from same RNA template isolated from zebra mussel feet were labelled with ALEXA 555 and 647 respectively. Then the two samples were hybridized to a single array slide. Two replicates were performed in this assay. The array data was analyzed with Limma and all the differentially expressed probesets were considered to be false positive results. It turned out that when we use *P *< 0.01 as selection criteria, only one false positive gene was identified no matter how big the fold change is. Therefore, *P *< 0.01 was used as selection standard in this study. The meaning of log (FC) when it was positive was explained in the legend of Table 1. The homologues of the genes are listed with the accession numbers and putative functions, as well as with the names of the species (Table 1). The up- or down-regulation of the factor to probes was decided by the coefficient. The dataset of this microarray study was deposited in Gene Expression Omnibus (GEO) with the series accession number GSE16397. Some of the identified genes with similar primary structures were differentiated by multiple sequence alignment with ClustalW [43]. The putative function of each identified gene was predicted based on the BLAST against the nucleotide database in GenBank.

### Microarray data validation

The one-step quantitative reverse transcription PCR (qRT-PCR) was performed with *Power *SYBR^® ^Green RNA-to-*C_T_*™ *1-Step *Kit (Applied Biosystems Inc., Foster City, CA) to identify the distribution of the candidate genes in zebra mussel tissues. The mussels were also collected from Vineyard Lake in Brooklyn, MI. The shells of randomly selected individuals were swabbed with 70% ethanol, followed by a soak in 150 ml sterilized double-distilled water containing 5,000 U penicillin, 5 mg streptomycin, and 10 mg gentamicin for 30 minutes [44]. Thereafter, the mussels were transferred to sterilized double distilled water. The RNA samples were extracted from hemocytes, feet, muscles, ctenidia, and mantles of zebra mussels. The protocol for hemocyte collection was described by Xu and Faisal in 2009 [45] and the other samples were taken by sterilized scissors and scalpels. The primers were designed by Primer Express™(Applied Biosystem Inc.). The primers used in this study are listed in Table 3.

**Table 3:** The gene specific primers used for qRT-PCR.

Target EST	Primer Name	Primer Sequence
AM229726	BG20A01_DPFP_RT_Fw	5'-ATG GGC CAT ATG ATA AGA AAC CA-3'
	BG20A01_DPFP_RT_Rv	5'-TCC AGG AGG TTC CAA TGG AA-3'
AM229885	BG13B05_EGP_RT_Fw	5'-CGG TTG CTA TAC ATG TGT CCA AGT-3'
	BG13B05_EGP_RT_Rv	5'-GGG AGG TTA CGG CGG CTA T-3'
AM230073	BG97/192B06_EGP_RT_Fw	5'-CAT CCC CGT ATG GGA TCC A-3'
	BG97/192B06_EGP_RT_Rv	5'-GGT GCA ACG GCC AAG TTT AT-3'
AM230384	BG17C09_NH_RT_Fw	5'-TCC GGA TAT TGG TTG TCC TCA T-3'
	BG17C09_NH_RT_Rv	5'-TTC TCC GTA GCC ACA CCA TTT-3'
*18SrRNA	*18SrRNA_Forward	5'-GAC ACG GCT ACC ACA TCC AA-3'
	*18SrRNA_Reverse	5'-CTC GAA AGA GTC CCG CAT TG-3'

* The primers used as internal reference for QRT-PCR.

The qRT-PCR was also applied to validate the expression profiles of the genes that were affected by the attachment status identified by microarray. The zebra mussels were allowed to fully attach themselves on glass Petri-dishes for six weeks. Then byssal threads from all individuals were cut off with scalpels; thereafter, five mussels were dissected and the RNA extracted from their feet was considered as group 0. The rest of the mussels were put back in the Petri-dishes in water, and allowed to regenerate their byssal threads. The RNA samples were extracted from the feet of the mussels at 12 hours, one day, two days, and three days post re-attachment. Two biological replicates were used for each group. Then for each candidate gene, the qRT-PCR was performed with these five group samples. The information of the gene specific RT Primers are listed in Table 3.

### RNA fluorescence in situ hybridization (FISH)

In order to locate the transcripts of two selected genes *in situ*, fluorescence *in situ *hybridization (FISH) was used. The zebra mussel foot tissue was fixed in 2% paraformaldehyde phosphate buffer saline (0.02 M NaH_2_PO_4_, 0.0077 M Na_2_HPO_4_, 1.4 M NaCl, 2% w/v paraformaldehyde, pH 8.0) for five hours at room temperature. The sample was then conserved in absolute ethanol until needed. The conserved sample was buried in paraffin and longitudinally sectioned at the same level (Figure 5 and 6). The five μm thick sectioned sample was obtained and put on an RNase- and DNase-free glass slide. Ten slides were obtained with one stained by H&E method.

The antisense RNA strand of a fragment of each gene was produced. Briefly, the PCR product of the gene fragment was cloned into pGEM^®^-T Easy Vector Systems (Promega U.S., Madison, WI). Then the positive colony was picked for sequencing with the M13 forward primer to clarify the direction of the insert of the recombinant plasmid. Once the direction of the insert was known, the T7 or Sp6 RNA polymerase was selected to synthesize the antisense RNA with the linearized recombinant plasmid. In this study, T7 polymerase was used on both probes. The probe DPFP-BG20_A01 was labeled with fluorescent dye Alexa 488 (Invitrogen) while the probe EGP-BG97/192_B06 was labeled with Alexa 594 (Invitrogen). The steps of the RNA synthesis and labeling can be found in the instructions of the two kits: FISH Tag™ RNA Green Kit (Invitrogen) and Fish Tag™ RNA Red Kit (Invitrogen). The RNA FISH was performed following the recommended protocol in the appendix of the Fish Tag™ RNA Kit instruction. The hybridization buffer mentioned in the protocol was replaced by ULTRAhybTM Ultrasensitive Hybridization Buffer (Applied Biosystems/Ambion, Austin, TX). The hybridized slides were visualized by Olympus BX41 (Olympus America Inc., Center Valley, PA) microscope under the excitation of a mercury lamp and the images were captured by Olympus DP25 digital camera (Olympus America Inc.). The image processing software DP2-BSW (Olympus America Inc.) was used to combine the different color channels into a single image.

## Authors' contributions

WX and MF designed the experiment and prepared the manuscript. WX conceived the experiment and data analyses. This manuscript has been reviewed and approved by both authors.

## Supplementary Material

Additional file 1 **The genes whose expression profiles have been significantly modified by the status of byssogenesis**. When Log (FC) > 0, the gene is up-regulated under the attachment status. * The differentially expressed ESTs with P < 0.01; ^A ^Also affected by Factor A (Temperature); ^B ^Also affected by Factor B (Agitation); ^C ^Also affected by Factor C (D.O.). Click here for file

Additional file 2 **The genes whose expression profiles have been significantly modified due to the change of the temperature**. Log (FC) > 0 means the gene is upregulated with the decrease of temperature. * The differentially expressed ESTs with *P *< 0.01; ^B ^Also affected by Factor B (Agitation); ^C ^Also affected by Factor C (D.O.); ^D ^Also affected by Factor D (Adhesion). Click here for file

Additional file 3 **The genes whose expression profiles have been significantly modified due to the change of D.O. level**. Log (FC) > 0 indicates the gene is upregulated when the dissolved oxygen in water is at lower level. * The differentially expressed ESTs with P < 0.01; ^A ^Also affected by Factor A (Temperature); ^B ^Also affected by Factor B (Agitation); ^D ^Also affected by Factor D (Adhesion). Click here for file

Additional file 4 **The genes whose expression profiles have been significantly modified due to the change of current frequency**. Log (FC) > 0 indicates the gene is upregulated when the mussel is under agitation. * The differentially expressed ESTs with P < 0.01; ^A ^Also affected by Factor A (Temperature); ^C ^Also affected by Factor C (D.O.); ^D ^Also affected by Factor D (Adhesion). Click here for file
