# Supplementary material for: Factorial microarray analysis of zebra mussel (Dreissena polymorpha: Dreissenidae, Bivalvia) adhesion
Source: BMC Genomics. 2010 May 28;11:341. doi: 10.1186/1471-2164-11-341 (PMC2894042; doi:10.1186/1471-2164-11-341)
Supplement: Additional file 1 — The genes whose expression profiles have been significantly modified by the status of byssogenesis. When Log (FC) > 0, the gene is up-regulated under the attachment status. * The differentially expressed ESTs with P < 0.01; A Also affected by Factor A (Temperature); B Also affected by Factor B (Agitation); C Also affected by Factor C (D.O.). [file 1471-2164-11-341-S1.DOC]

## Additional file 1. The genes whose expression profiles have been significantly modified by the status of byssogenesis.

| **Gene ID** | **Accession #** | **p.value** | **Log (FC)** | **Homologue** |
| --- | --- | --- | --- | --- |
| **BG17_G02* ^B^** | AM230231 | 0.00011 | -0.6 | N/A |
| **BG10_C05*** | AM229883 | 0.00114 | 0.146 | AAV80789.1| Excretory salivary gland peptide [*Ixodes scapularis*] |
| **BG33_H03*** | AM230185 | 0.0013 | -0.168 | N/A |
| **BG97/192_B06* ^C^** | AM230076 | 0.00317 | 0.214 | AAS92593.1| Excretory/secretory protein Juv-p120 precursor [*Litomosoides sigmodontis*] |
| **BG20_F04*** | AM230089 | 0.00339 | -0.122 | N/A |
| **BG13_F10*** | AM230013 | 0.00374 | -0.130 | AAV80789.1| Excretory gland peptide [*Ixodes scapularis*] |
| **BG23_B03* ^A^** | AM229897 | 0.00498 | -0.184 | AAV80789.1| Excretory gland peptide [*Ixodes scapularis*] |
| **BG14_A11*** | AM230170 | 0.00523 | 0.272 | ABI52762.1| 60S ribosomal protein L27 [*Argas monolakensis*] |
| **BG16_D03* ^C^** | AM230168 | 0.00801 | 0.168 | AAN05585.1| Ribosomal protein L22 [*Argopecten irradians*] |
| **BG13_C11*** | AM230205 | 0.00946 | -0.124 | N/A |
| **BG34_H01*** | AM229894 | 0.01029 | 0.174 | AAV80789.1| Excretory salivary gland peptide [*Ixodes scapularis*] |
| **BG20_A01** | AM229724 | 0.01149 | 0.370 | AF265353_1| Byssal protein Dpfp1 precursor [Dreissena polymorpha] |
| **BG26_C06** | AM230431 | 0.0119 | -0.158 | N/A |
| **BG05_D06** | AM230302 | 0.01191 | 0.152 | N/A |
| **BG17_C09 ^A^** | AM230384 | 0.01206 | 0.316 | N/A |
| **BG08_B10** | AM230189 | 0.01278 | 0.096 | N/A |
| **BG10_F10** | AM230328 | 0.01321 | -0.152 | N/A |
| **BG32_C03** | AM229736 | 0.01371 | -0.282 | AAC39039.1| Foot protein 1 precursor [*Dreissena polymorpha*] |
| **BG32_D04** | AM230156 | 0.01374 | 0.116 | BAB12683.1| Polypeptide release factor 3 [*Yarrowia lipolytica*] |
| **BG15_D07** | AM230362 | 0.01379 | -0.110 | N/A |
| **BG23_E03** | AM229779 | 0.01382 | -0.114 | BAE93436.1| Shematrin-4 [*Pinctada fucata*] |
| **BG13_B05^C^** | AM229816 | 0.0139 | 0.300 | AAV80789.1| Excretory gland peptide [*Ixodes scapularis*] |
| **BG23_C05** | AM230080 | 0.01572 | -0.116 | N/A |
| **BG14_C07 ^C^** | AM230353 | 0.01679 | 0.192 | N/A |
| **BG22_C12** | AM229893 | 0.01706 | -0.144 | N/A |
| **BG07_F07** | AM230102 | 0.01764 | -0.114 | NP_504109.1| Neuropeptide-Like protein nlp-29 [*Caenorhabditis elegans*] |
| **BG06_E09** | AM230224 | 0.01777 | -0.146 | AAF75279.1| Byssal protein Dpfp1 precursor [*Dreissena polymorpha*] |
| **BG04_A09** | AM230090 | 0.02014 | -0.108 | N/A |
| **BG12_D10** | AM230249 | 0.02035 | 0.292 | N/A |
| **BG31_E11 ^C^** | AM230254 | 0.02171 | 0.294 | N/A |
| **BG25_H08** | AM230042 | 0.02211 | 0.316 | N/A |
| **BG04_G04** | AM230066 | 0.02267 | -0.100 | ABD62888.1| Serine proteinase-like protein [*Penaeus monodon*] |
| **BG04_F03** | AM230188 | 0.02308 | -0.322 | N/A |
| **MF030105_B09** | AM230050 | 0.02341 | -0.152 | N/A |
| **BG28_H05 ^A^** | AM229934 | 0.02359 | 0.228 | BAE93436.1| Shematrin-4 [*Pinctada fucata*] |
| **BG34_F03** | AM230174 | 0.02459 | 0.140 | ABG81984.1| Ribosomal protein S14e [*Diaphorina citri*] |
| **BG33_E04** | AM229997 | 0.02496 | -0.134 | N/A |
| **BG18_B07** | AM230216 | 0.0271 | 0.122 | N/A |
| **BG10_D04** | AM230104 | 0.02748 | -0.286 | AAK68690.1| Hemicentin [*Homo sapiens*] |
| **BG20_B09** | AM230000 | 0.02829 | -0.114 | AAV80789.1| Excretory salivary gland peptide [*Ixodes scapularis*] |
| **BG30_H12** | AM229764 | 0.02853 | -0.106 | N/A |
| **BG25_F07** | AM229895 | 0.0296 | 0.116 | AAV80789.1| Excretory salivary gland peptide [*Ixodes scapularis*] |
| **MF030105_G10** | AM229731 | 0.02971 | 0.326 | AAF75279.1| Byssal protein Dpfp1 precursor [*Dreissena polymorpha*] |
| **BG08_B08** | AM230321 | 0.0341 | 0.218 | N/A |
| **MF030105_H09** | AM229864 | 0.03422 | -0.082 | AAV80789.1| Excretory salivary gland peptide [*Ixodes scapularis*] |
| **BG06_C03** | AM229866 | 0.03508 | -0.284 | N/A |
| **BG23_B06** | AM229738 | 0.03609 | 0.092 | AF265353_1 Byssal protein Dpfp1 precursor [*Dreissena polymorpha*] |
| **BG05_E08** | AM230198 | 0.03709 | 0.114 | N/A |
| **MF030105_F07** | AM230273 | 0.03711 | -0.094 | N/A |
| **BG26_B09 ^A^** | AM229855 | 0.03759 | -0.096 | N/A |
| **MF030105_C07 ^B^** | AM229749 | 0.03772 | -0.290 | N/A |
| **BG34_B04** | AM230256 | 0.03796 | 0.086 | N/A |
| **MF030105_F02** | AM230272 | 0.03899 | -0.220 | N/A |
| **BG31_A11** | AM230107 | 0.04079 | 0.094 | EAY59350.1| Serine protease pepD [*Mycobacterium tuberculosis* C] |
| **BG03_B01^A^** | AM229901 | 0.04086 | 0.128 | AAV80789.1| Excretory salivary gland peptide [*Ixodes scapularis*] |
| **BG05_B04 ^A^** | AM230070 | 0.04134 | 0.110 | N/A |
| **BG10_B09** | AM230213 | 0.0415 | 0.210 | N/A |
| **BG97/192_A06** | AM230225 | 0.04155 | 0.140 | N/A |
| **BG08_F02** | AM229725 | 0.04254 | 0.104 | AAF75279.1| Byssal protein Dpfp1 precursor [*Dreissena polymorpha*] |
| **BG16_A07** | AM230120 | 0.04305 | -0.120 | ABE03741.1| Prophenoloxidase activating factor [*Penaeus monodon*] |
| **BG26_G12** | AM229957 | 0.04376 | -0.098 | AAV80789.1| Excretory salivary gland peptide [*Ixodes scapularis*] |
| **BG04_A11** | AM230155 | 0.04379 | 0.100 | BAB12683.1| Polypeptide release factor 3 [*Yarrowia lipolytica*] |
| **BG29_E08** | AM229745 | 0.04467 | -0.124 | AAF75279.1|Byssal protein Dpfp1 precursor [*Dreissena polymorpha*] |
| **BG14_D01** | AM230121 | 0.04495 | -0.086 | N/A |
| **BG13_A02** | AM230345 | 0.04566 | 0.174 | N/A |
| **BG04_H02** | AM229815 | 0.04732 | -0.100 | AAT92111.1| Excretory gland peptide NPL-2 [*Ixodes pacificus*] |
| **BG14_D12** | AM229862 | 0.04786 | 0.090 | BAE93436.1| Shematrin-4 [*Pinctada fucata*] |
| **BG15_F03** | AM229727 | 0.04786 | 0.096 | AAF75279.1|Byssal protein Dpfp1 precursor [*Dreissena polymorpha*] |
| **BG17_F03** | AM230143 | 0.04871 | -0.258 | N/A |
| **BG10_H04** | AM230118 | 0.04908 | 0.138 | N/A |
| **BG31_D01** | AM230039 | 0.04942 | 0.266 | NP_505834.1| Neuropeptide-Like protein nlp-33 [*Caenorhabditis elegans*] |
| **BG15_D03** | AM229780 | 0.04982 | 0.194 | N/A |
| **BG30_D12 ^C^** | AM229805 | 0.04991 | -0.090 | BAE93436.1| Shematrin-4 [*Pinctada fucata*] |

When Log (FC) > 0, the gene is up-regulated under the attachment status.

* The differentially expressed ESTs with *P* <0.01.

^A^ Also affected by Factor A (Temperature); ^B^ Also affected by Factor B (Agitation);

^C^ Also affected by Factor C (D.O.).
