# Supplementary material for: Factorial microarray analysis of zebra mussel (Dreissena polymorpha: Dreissenidae, Bivalvia) adhesion
Source: BMC Genomics. 2010 May 28;11:341. doi: 10.1186/1471-2164-11-341 (PMC2894042; doi:10.1186/1471-2164-11-341)
Supplement: Additional file 2 — The genes whose expression profiles have been significantly modified due to the change of the temperature. Log (FC) > 0 means the gene is upregulated with the decrease of temperature. * The differentially expressed ESTs with P < 0.01; B Also affected by Factor B (Agitation); C Also affected by Factor C (D.O.); D Also affected by Factor D (Adhesion). [file 1471-2164-11-341-S2.DOC]

## Additional file 2. The genes whose expression profiles have been significantly modified due to the change of the temperature.

| **Genes ID** | **Accession #** | **p.value** | **Log(FC)** | **Homologues** |
| --- | --- | --- | --- | --- |
| **BG17_C09*^D^** | AM230384 | 7.00E-05 | 0.628 | N/A |
| **BG27_B08*** | AM230019 | 0.00043 | 0.232 | AAT92111.1| Excretory gland peptide NPL-2 [*Ixodes pacificus*] |
| **BG28_H05* ^D^** | AM229934 | 0.0052 | -0.232 | BAE93436.1| Shematrin-4 [*Pinctada fucata*] |
| **BG03_B01*** | AM229901 | 0.00653 | -0.196 | AAV80789.1| Excretory gland peptide [*Ixodes scapularis*] |
| **BG23_D02*** | AM229752 | 0.0068 | -0.190 | AAV80789.1| Excretory gland peptide [*Ixodes scapularis*] |
| **BG28_C09** | AM229790 | 0.01034 | -0.254 | BAE93436.1| shematrin-4 [*Pinctada fucata*] |
| **BG17_F07** | AM229867 | 0.01123 | -0.114 | BAE93436.1| shematrin-4 [*Pinctada fucata*] |
| **BG27_H05** | AM230068 | 0.01216 | 0.182 | AAC05725.1| RNA helicase A [*Mus musculus*] |
| **BG27_E09** | AM229903 | 0.01427 | 0.200 | AAV80789.1| Excretory gland peptide [*Ixodes scapularis*] |
| **BG26_D08** | AM230432 | 0.01818 | 0.108 | AAV80789.1| putative secreted salivary gland peptide [*Ixodes scapularis*] |
| **BG13_B06** | AM229798 | 0.01919 | 0.302 | AAT92111.1| Excretory gland peptide NPL-2 [*Ixodes pacificus*] |
| **BG28_B04** | AM230435 | 0.02361 | -0.380 | N/A |
| **BG29_E12** | AM230154 | 0.02449 | -0.130 | N/A |
| **BG05_B04** | AM230070 | 0.02598 | -0.134 | N/A |
| **BG16_F01** | AM230081 | 0.02693 | 0.192 | AAS92593.1| Excretory/secretory protein Juv-p120 precursor [*Litomosoides sigmodontis*] |
| **BG23_B03 ^D^** | AM229897 | 0.02749 | 0.154 | NP_505834.1|Neuropeptide-Like protein nlp-33 [*Caenorhabditis elegans*] |
| **BG26_C05** | AM229917 | 0.02753 | 0.160 | AAV80789.1| Excretory gland peptide [*Ixodes scapularis*] |
| **BG18_D01** | AM229730 | 0.02863 | 0.402 | N/A |
| **BG16_G06** | AM230258 | 0.03025 | -0.424 | N/A |
| **BG05_A11** | AM229750 | 0.03037 | 0.138 | Q25460| Adhesive plaque matrix protein [*Mytilus edulis*] |
| **BG27_B10** | AM230153 | 0.03739 | 0.130 | BAB12683.1| Polypeptide release factor 3 [*Yarrowia lipolytica*] |
| **BG33_A08** | AM229799 | 0.03749 | 0.186 | AAT92111.1| Excretory gland peptide NPL-2 [*Ixodes pacificus*] |
| **BG97/192_D02** | AM229740 | 0.03991 | -0.192 | N/A |
| **BG07_H06 ^B^** | AM230138 | 0.04305 | 0.156 | ABN13415.1| Choriogenin H [*Oryzias melastigma*] |
| **BG28_E01 ^C^** | AM229772 | 0.04418 | 0.106 | N/A |
| **BG28_F03** | AM230145 | 0.04425 | 0.138 | N/A |
| **BG26_B09 ^D^** | AM229879 | 0.0449 | 0.102 | N/A |

Log (FC) > 0 means the gene is upregulated with the decrease of temperature.

* The differentially expressed ESTs with *P* <0.01.

^B^ Also affected by Factor B (Agitation); ^C^ Also affected by Factor C (D.O.);

^D^ Also affected by Factor D (Adhesion).
