# Supplementary material for: Factorial microarray analysis of zebra mussel (Dreissena polymorpha: Dreissenidae, Bivalvia) adhesion
Source: BMC Genomics. 2010 May 28;11:341. doi: 10.1186/1471-2164-11-341 (PMC2894042; doi:10.1186/1471-2164-11-341)
Supplement: Additional file 3 — The genes whose expression profiles have been significantly modified due to the change of D.O. level. Log (FC) > 0 indicates the gene is upregulated when the dissolved oxygen in water is at lower level. * The differentially expressed ESTs with P < 0.01; A Also affected by Factor A (Temperature); B Also affected by Factor B (Agitation); D Also affected by Factor D (Adhesion). [file 1471-2164-11-341-S3.DOC]

## Additional file 3. The genes whose expression profiles have been significantly modified due to the change of D.O. level.

| **Gene ID** | **Accession #** | **p.value** | | **Log (FC)** | | **Homologue** |
| --- | --- | --- | --- | --- | --- | --- |
| **BG23_D06*** | AM230109 | | 0.0026 | | -0.270 | N/A |
| **BG14_F09*** | AM230247 | | 0.00639 | | 0.160 | N/A |
| **BG12_B10** | AM230219 | | 0.01343 | | 0.196 | N/A |
| **BG30_D12 ^D^** | AM229805 | | 0.0138 | | 0.096 | BAE93436.1| Shematrin-4 [*Pinctada fucata*] |
| **MF030105_D04** | AM230269 | | 0.01606 | | -0.250 | N/A |
| **BG18_B03** | AM230397 | | 0.02033 | | 0.234 | N/A |
| **BG16_D03 ^D^** | AM230168 | | 0.02521 | | 0.112 | AAN05585.1| Ribosomal protein L22 [*Argopecten irradians*] |
| **BG05_D02** | AM230211 | | 0.02647 | | 0.206 | N/A |
| **BG14_C07 ^D^** | AM230353 | | 0.02743 | | 0.142 | N/A |
| **BG97/192_C05** | AM230277 | | 0.02815 | | 0.126 | N/A |
| **BG13_E01** | AM229847 | | 0.02927 | | 0.088 | BAE93436.1| Shematrin-4 [*Pinctada fucata*] |
| **BG25_H11 ^B^** | AM230248 | | 0.02979 | | -0.094 | N/A |
| **BG16_B01** | AM229753 | | 0.03044 | | 0.104 | AAV80789.1| Excretory gland peptide [*Ixodes scapularis*] |
| **MF030105_H01** | AM229821 | | 0.03044 | | 0.268 | AAT92111.1| Excretory gland peptide NPL-2 [*Ixodes pacificus*] |
| **BG07_F03** | AM229726 | | 0.03069 | | -0.166 | AAF75279.1| Byssal protein Dpfp1 precursor [*Dreissena polymorpha*] |
| **BG12_G10** | AM230123 | | 0.03203 | | 0.108 | N/A |
| **BG97/192_B06 ^D^** | AM230076 | | 0.03528 | | 0.118 | AAS92593.1| Excretory/secretory protein Juv-p120 precursor [*Litomosoides sigmodontis*] |
| **BG27_F10** | AM230146 | | 0.04161 | | 0.094 | N/A |
| **BG24_B11** | AM230213 | | 0.04259 | | 0.092 | N/A |
| **BG28_E01 ^A^** | AM229772 | | 0.04306 | | 0.078 | N/A |
| **BG26_B01** | AM230172 | | 0.04342 | | -0.082 | AAK95191.1| 40S ribosomal protein S9 [*Ictalurus punctatus*] |
| **BG31_E11 ^D^** | AM230254 | | 0.04511 | | -0.206 | N/A |
| **BG13_B05 ^D^** | AM229885 | | 0.04647 | | 0.192 | AAV80789.1| Excretory salivary gland peptide [*Ixodes scapularis*] |
| **BG22_H06 ^B^** | AM229789 | | 0.04694 | | -0.078 | N/A |
| **BG20_F01** | AM229782 | | 0.04702 | | -0.214 | N/A |
| **BG25_B01** | AM229884 | | 0.04732 | | 0.178 | AAV80789.1| Excretory salivary gland peptide [*Ixodes scapularis*] |

Log (FC) > 0 indicates the gene is upregulated when the dissolved oxygen in water is at lower level.

* The differentially expressed ESTs with P <0.01.

^A^ Also affected by Factor A (Temperature); ^B^ Also affected by Factor B (Agitation);

^D^ Also affected by Factor D (Adhesion).
