# Supplementary material for: Factorial microarray analysis of zebra mussel (Dreissena polymorpha: Dreissenidae, Bivalvia) adhesion
Source: BMC Genomics. 2010 May 28;11:341. doi: 10.1186/1471-2164-11-341 (PMC2894042; doi:10.1186/1471-2164-11-341)
Supplement: Additional file 4 — The genes whose expression profiles have been significantly modified due to the change of current frequency. Log (FC) > 0 indicates the gene is upregulated when the mussel is under agitation. * The differentially expressed ESTs with P < 0.01; A Also affected by Factor A (Temperature); C Also affected by Factor C (D.O.); D Also affected by Factor D (Adhesion). [file 1471-2164-11-341-S4.DOC]

## Additional File 4. The genes whose expression profiles have been significantly modified due to the change of current frequency.

| **Gene ID** | **Accession #** | | **p.value** | | **Log (FC)** | **Homologue** |
| --- | --- | --- | --- | --- | --- | --- |
| **BG29_B09*** | | AM230251 | 0.00415 | 0.232 | | N/A |
| **BG22_H06*^C^** | | AM229789 | 0.00952 | -0.156 | | N/A |
| **BG10_F01** | | AM230379 | 0.01195 | 0.148 | | N/A |
| **BG29_A05** | | AM230250 | 0.02185 | 0.106 | | N/A |
| **MF030105_C07 ^D^** | | AM229749 | 0.02546 | -0.376 | | N/A |
| **BG07_H06 ^A^** | | AM230138 | 0.0265 | 0.188 | | ABN13415.1| Choriogenin H [*Oryzias melastigma*] |
| **BG17_G02 ^D^** | | AM230231 | 0.03856 | 0.332 | | N/A |
| **BG25_H11 ^C^** | | AM230248 | 0.03927 | -0.130 | | N/A |
| **BG12_H05** | | AM230222 | 0.0491 | 0.152 | | N/A |

Log (FC) > 0 indicates the gene is upregulated when the mussel is under agitation.

* The differentially expressed ESTs with P <0.01.

^A^ Also affected by Factor A (Temperature); ^C^ Also affected by Factor C (D.O.);

^D^ Also affected by Factor D (Adhesion).
